# Correlation of Hemodynamic and Respiratory Parameters in Invasive Cardiopulmonary Exercise Testing (iCPET)

**DOI:** 10.3390/life12050655

**Published:** 2022-04-28

**Authors:** Dirk Habedank, Anne Obst, Alexander Heine, Beate Stubbe, Ralf Ewert

**Affiliations:** 1 Clinical Medicine Department of Cardiology, DRK Kliniken Berlin Köpenick, D 12559 Berlin, Germany; 2 Clinical Medicine Department of Pneumology, University Medicine Greifswald, D 17475 Greifswald, Germany; anne.obst@med.uni-greifswald.de (A.O.); alexander.heine@med.uni-greifswald.de (A.H.); beate.stubbe@uni-greifswald.de (B.S.); ralf.ewert@med.uni-greifswald.de (R.E.)

**Keywords:** invasive cardiopulmonary exercise testing, right heart catheter, cardiac output, cardiac index, pulmonary arterial pressure, ventilation, oxygen uptake

## Abstract

Background: Invasive cardiopulmonary exercise testing (iCPET) is an integral part in the advanced diagnostic workup of pulmonary hypertension (PH). Our study evaluated the relation between hemodynamic and respiratory parameters at two different resting conditions and two defined low exercise levels with a close synchronization of measurements in a broad variety of dyspnea patients. Subjects and methods: We included 146 patients (median age 69 years, range 22 to 85 years, *n* = 72 female) with dyspnea of uncertain origin. Invasive hemodynamic and gas exchange parameters were measured at rest, 45° upright position, unloaded cycling, 25 and 50 W exercise. All measurements were performed in a single RHC procedure. Results: Oxygen uptake (VO${}_{2}$/body mass) correlated significantly with cardiac index (all *p* ≤ 0.002) at every resting and exercise level and with every method of cardiac output measurement (thermodilution, method of Fick). Mean pulmonary arterial pressure (PAPmean) correlated with all respiratory parameters (respiratory rate, partial end-tidal pressures of oxygen and carbon dioxide [petCO${}_{2}$ and petO${}_{2}$], ventilation/carbon dioxide resp. oxygen ratio [VE/VCO${}_{2}$, VE/VO${}_{2}$], and minute ventilation [VE], all *p* < 0.05). These correlations improved with increasing exercise levels from rest via unloaded cycling to 25 W. There was no correlation with right atrial or pulmonary arterial wedge pressure. Summary: In dyspnea patients of different etiologies, the cardiac index is closely linked to VO${}_{2}$ at every level of rest and submaximal exercise. PAPmean is the only pressure that correlates with different respiratory parameters, but this correlation is highly significant and stable at rest, unloaded cycling and at 25 W.

## 1. Introduction

The invasive measurement of hemodynamic parameters by right heart catheterisation (RHC) is the decisive diagnostic method in pulmonary hypertension (PH). It is mandatory for therapeutic decisions and allows a stratification of the patients’ prognosis. The indication, procedure and interpretation of RHC have been standardized to a large extent, for instance by the European Society of Cardiology in 2017 [1]. Cardiopulmonary exercise testing (CPET) evaluates different aspects of pathophysiology, namely regarding exercise, and is, in contrast to RHC, a non-invasive method. Exercise limitation and response to therapy in pulmonary arterial hypertension (PAH) can be graded by CPET [2,3]. As with RHC, the application and performance of CPET in PH were subject of a standardization process, and expert consensus has been published [4,5]. The combination of both methods is termed “invasive cardiopulmonary exercise testing” (iCPET) and provides a very differentiated analysis of the etiology of dyspnea and the response to exercise. It has been shown to predict prognosis in patients with chronic systolic heart failure [6], heart failure with preserved ejection fraction (HFpEF) [7], and with exertional dyspnea in general [8]. iCPET is an integral part in the advanced diagnostic workup of PH [9].

One must consider that iCPET with repeated measurement of thermodilution is difficult under exercise conditions and requires a trained and well-cooperating staff. This might be one of the reasons why the mode of exercise for iCPET is not well standardized [5], despite the several proposals that have been published in recent years [9,10,11]. Aside from the still necessary standardization, the allocation of RHC parameters to respiratory parameters at exercise was mostly evaluated in selected groups of patients and at various levels of exercise, i.e., at unloaded cycling, anaerobic threshold, 25 W or peak exercise. About 30 years ago, early studies found a significant relation between the respiratory parameters VE/VCO${}_{2}$-slope and peak oxygen uptake (peakVO${}_{2}$) and the invasive measured hemodynamic parameters cardiac index (CI), mean pulmonary artery pressure (PAPmean), and pulmonary arterial wedge pressure (PAWP), but all correlations still referred to patients with chronic heart failure [12]. By contrast, a pilot study using a micro-manometer-tipped catheter in patients with PAH at exercise showed that ventilatory equivalents for oxygen (VE/VO${}_{2}$) and carbon dioxide (VE/VCO${}_{2}$) correlated with PAPmean at exercise, but oxygen pulse and oxygen uptake (VO${}_{2}$) did not [13].

Most parameters have been evaluated at rest and at peak exercise only, and the above-mentioned technical and logistical demands might have prevented a stepwise analysis. Against this background, our study evaluated the relationship between hemodynamic and respiratory parameters at two different resting conditions and two defined low exercise levels. We defined a protocol that was aimed at the greatest possible synchronization of measurements and applied the method to a broad variety of dyspnea patients.

## 2. Patients and Methods

### 2.1. Patients

This single-centre retrospective study recruited from a total of 306 patients that were referred to the Greifswald University hospital between 2016 and 2020 due to dyspnea of uncertain etiology. All of these patients underwent right heart catheterization during exercise with measurement of respiratory parameters (iCPET). We performed these procedures with predefined and close synchronization of thermodilution, blood gas sampling and measurement of respiratory parameters. In case of multiple testing of patients in long-time follow-up, only the first iCPET was included, so that the final study population consisted of 146 patients. The study was approved by the Ethics Committee of Greifswald University (No. BB 215/20 of 17 November 2020, which was amended on 28 January 2021. All patients gave their written informed consent prior to the procedure.

### 2.2. Right Heart Catheterization

The indication for exercise RHC with simultaneous measurement of oxygen uptake was the suspicion of a multi-factorial etiology of dyspnea as a result of the preceding non-invasive examinations. The latter included ECG, X-ray of the thorax, echocardiography, CPET, and bodplethysmography. All catheter procedures were performed in hospitalized patients and followed the guidelines of the ESC/ERS [14] and German recommendations [15]. We have published the detailed course of the exercise RHC previously [16]. In brief, this procedure starts with a hemodynamic measurement at rest in a supine position (labelled in the following “baseline”) and a second measurement at rest in a semi-supine position (“45° at rest”), followed by unloaded cycling in a semi-supine position for 3 to 5 min (“unloaded cycling”), and 3 to 5 min cycling at 25 W. If both anaerobic threshold (in the literature, synonymously “first ventilatory threshold”) was not reached and dyspnea reported was only mild to moderate, a 50 W step was appended. On average, this was 59% of the peak VO${}_{2}$ that the patient achieved on a cycle ergometer 3 to 5 days before. This pre-test was performed in a manner that was symptom limited, in a sitting position and without RHC. At rest we measured cardiac output (CO), respectively CI, by thermodilution (TD) and the direct and indirect method of Fick (dFM resp. iFM, with the iFM method using the table of LaFarge [17]). At exercise, CO was calculated at every level by two methods in each patient: by TD using the mean of 3 (up to 5) cold water injections and verification of the visualized temperature curve, and simultaneously by the dFM method. The latter was performed after reaching a steady state (mostly after 3–5 min) of VO${}_{2}$ at the respective exercise level. PH was defined according to the definition at time of study enrolment as PAPmean ≥ 25 mmHg, and pulmonary arterial hypertension (PAH) was defined as PAPmean ≥ 25 mmHg plus PAWP < 15 mmHg. The recent definition with a cutoff at PAPmean >20 mmHg and inclusion of pulmonary vascular resistance (PVR) ≥ 3 Wood units (≥240 dyn·s·cm${}^{-5}$) [18] was published in 2019 and therefore not applicable for our study that started in 2016.

### 2.3. Cardiopulmonary Exercise Test and Blood Gas Analysis

**Figure 1:**
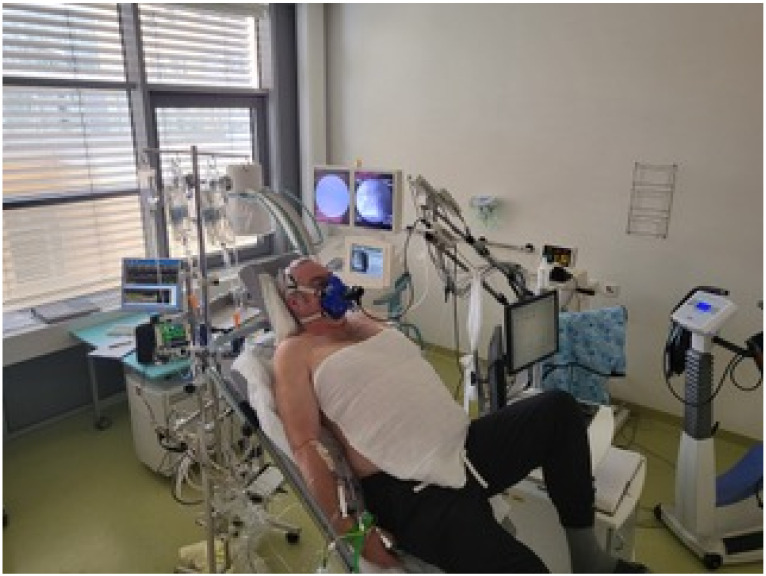
Invasive cardiopulmonary exercise testing at Greifswald university. Typical assessment situation with patient in a 45° upright position on an ergometer, wearing a Rudolph mask. Right heart catheter via vena cephalica, pressure transformer below ergometer, fluoroscopy (background middle), pressure monitor and analysis (background left), gas analyzer and metabolic card (above and behind left knee).

VO${}_{2}$ and VCO${}_{2}$ were measured with a 10 s average by a cardiopulmonary exercise test system (SentrySuite; Viasys Healthcare GmbH, Höchberg, Germany) via a face mask at room air. Blood samples for the measurement of mixed-venous and arterial oxygen saturation and arterial partial pressures of oxygen (p${}_{a}$O${}_{2}$) and carbondioxide (p${}_{a}$CO${}_{2}$) were taken at the same time as the exhaled gas measurement and within a 30 s frame of the cold water injection for TD measurement. Blood gases were analyzed immediately (ABL 90; Radiometer, Copenhagen, Denmark). A marker was set at the exact time of blood sampling in the electronic file, and CPET data were extracted later from the measurement data file at this very point and 10 s before and 10 s after. In order to avoid outliers by very deep or shallow breathing on the one hand and to be very close to hemodynamic measurement on the other hand, we transferred the −10 s/0/+10 s datasets to a database program and calculated the mean for each respiratory parameter. These data were analyzed statistically as described below. The situation of the assessment is shown in Figure 1. All data were acquired, calculated and saved electronically.

### 2.4. Statistics

Nominal data are given in absolute numbers and percent, and continuous data are given as median (25th; 75th percentile). Pearson and Spearman correlation coefficients (r) were computed to assess the relationships between gas exchange and hemodynamic measures at rest and at different levels of exercise. A value of *p* < 0.05 was considered statistically significant. The analyses were performed with the SAS 9.4 program (SAS Institute Inc., Cary, NC, USA).

## 3. Results

### 3.1. Patient Characteristics

Nearly half of the *n* = 146 patients were female (*n* = 72; 49.3%), and the median age was 69 (25th to 75th percentile, 59–76) years. The median body mass index was 27.2 (25th/75th percentile, 24.1 to 32.4) kg/m${}^{2}$. The most frequent diagnoses leading to RHC indication were HFpEF (*n* = 44), connective tissue diseases including systemic sclerosis (*n* = 29), pulmonary embolism (*n* = 20), pulmonary fibrosis including sarcoidosis (*n* = 5), COPD (*n* = 5), and heart failure with reduced ejection fraction (HFrEF, *n* = 5). All others diagnoses concerned less than five patients each and are fully listed in Table 1.

According to the hemodynamic measurements at rest, *n* = 83 (56.8%) of the patients had pulmonary hypertension. These patients were subdivided according to the Nice classification into class 1: *n* = 30, class 2: *n* = 34, class 3: *n* = 8, class 4: *n* = 10, and class 5: *n* = 1. The proportion of main concomitant diseases was (in n; %): arterial hypertension (97; 66.4%), coronary heart disease (37; 25.3%), diabetes mellitus (42; 28.8%), atrial fibrillation (45; 30.8%), venous thromboembolism (41; 28.8%), chronic renal failure (24; 16.4%), and history of cancer (26; 17.8%). Of all 146 patients, only 1 had a procedure-related complication with a small pneumothorax that absorbed spontaneously within 2 days. Baseline demographic data and functional parameters are shown in Table 1.

142 patients underwent a symptom-limited cardiopulmonary exercise without RHC 3 to 5 days before iCPET. These tests showed a reduced exercise capacity (peakVO${}_{2}$ in % of predicted = 64.7 ± 19.6%) and an impaired ventilatory efficiency (VE/VCO${}_{2}$-slope = 42.4 ± 14.3). The utilization of ventilatory reserve (VE/MVV) was 61.1 ± 16.1, and 13 patients (9.5%) had a pathological exhaustion (VE/MVV > 80%). The mean power at maximum exercise was 81.4 ± 40.5 W. Data of this CPET are also presented in Table 1. Reference values for pulmonary function were taken from [19], and reference values for CPET parameters were taken from [20].

### 3.2. Oxygen Uptake and Hemodynamic Parameters

**Figure 2:**
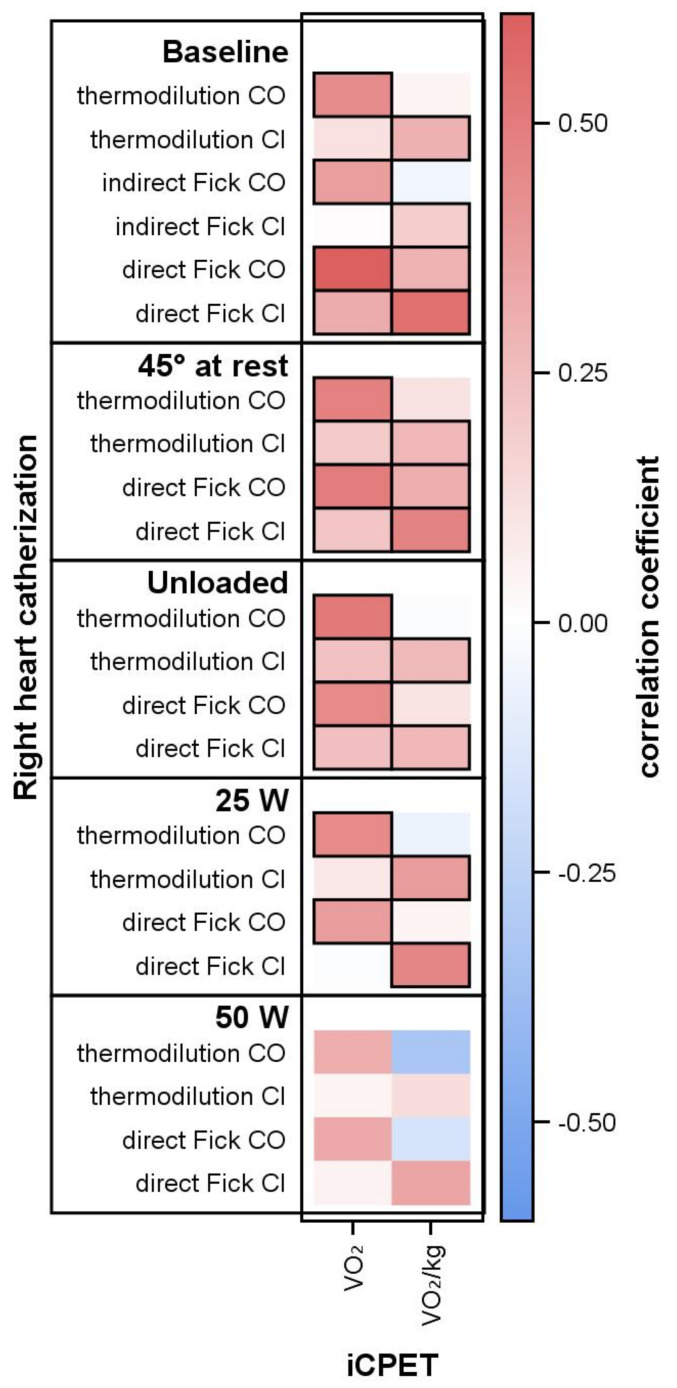
Cardiac output, oxygen uptake and correlation coefficients. Oxygen uptake in milliliters per minute (VO${}_{2}$) and per kg body weight (VO${}_{2}$/kg). Thick frames indicate significance level *p* < 0.05.

Oxygen uptake VO${}_{2}$/body weight (in mL/min/kg) correlated significantly with cardiac index (all *p* ≤ 0.002). This correlation was very robust because it was reproducible at every level, i.e., at rest (supine and 45°), at unloaded cycling and at 25 W. The correlation between CI and VO${}_{2}$/body weight was proven for every method of hemodynamic measurement, with somewhat lower correlation coefficients for CI by TD (r ≤ 0.37; *p* < 0.001) and the best correlation for the dFM (r ≤ 0.54; *p* < 0.001). The absolute, i.e., not body weight-related, values of VO${}_{2}$ (in mL/min) correlated with cardiac output and with lower correlation coefficients also with CI at rest, 45° and unloaded cycling (Figure 2). A special pattern was proven at 25 W with correlations only between the body-weight-related (VO${}_{2}$/body weight and CI) and body-weight-unrelated (VO${}_{2}$ and CI) parameters. All correlations lost significance at 50 W, in parallel with the lower number of patients.

### 3.3. Respiratory Parameters and PAPmean, PAWP and PVR

**Figure 3:**
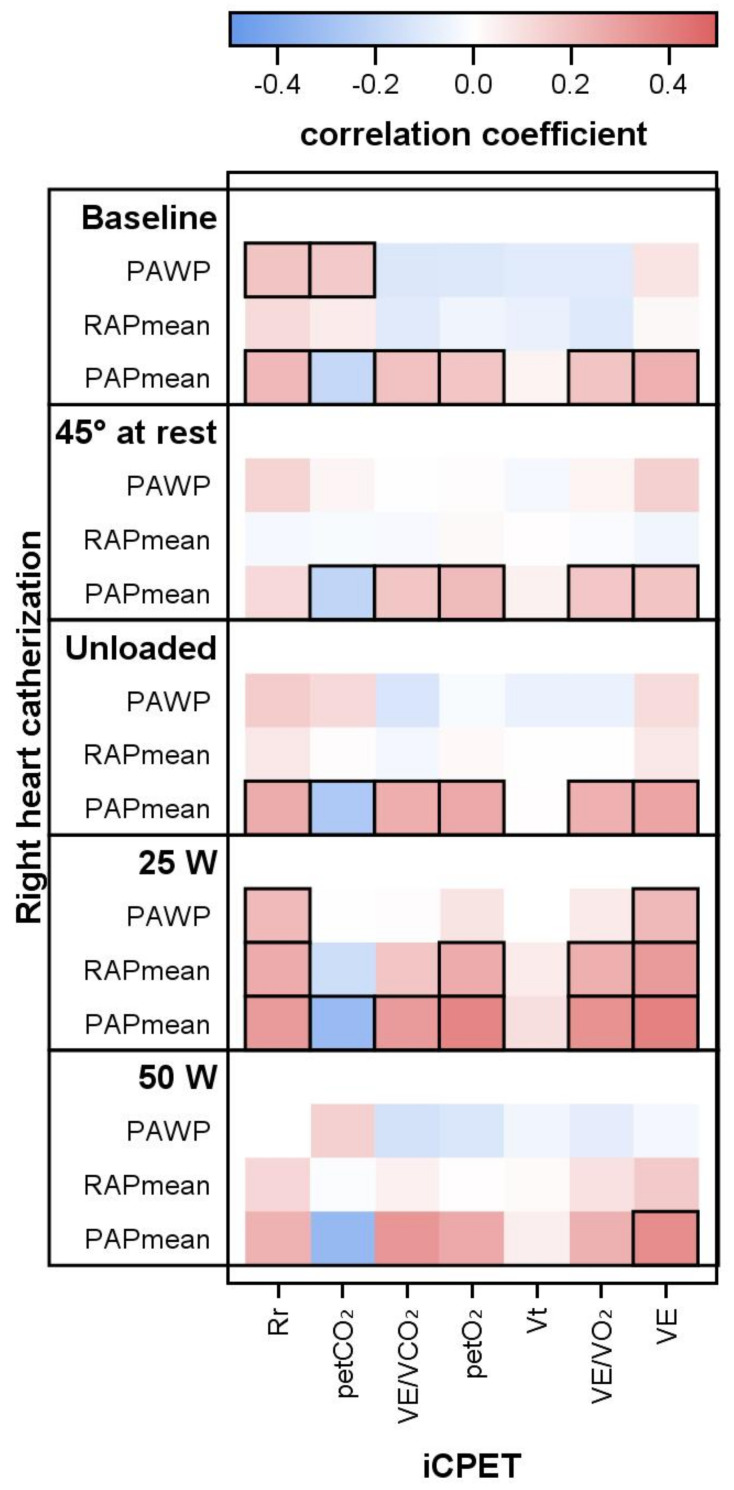
Right heart pressures, respiratory values and correlations. PAWP, pulmonary arterial wedge pressure; RAPmean mean, right arterial pressure; PAPmean, mean pulmonary arterial pressure; Rr, respiratory rate; p${}_{\mathrm{et}}$CO${}_{2}$, end-tidal partial pressure of carbon dioxide; p${}_{\mathrm{et}}$O${}_{2}$, end-tidal partial pressure of oxygen; VE/VCO${}_{2}$, ventilation-to-carbon-dioxide output ratio; VE/VO${}_{2}$, ventilation-to-oxygen-uptake ratio; VE, ventilation per minute; V${}_{t}$, tidal volume. Thick frames indicate significance level *p* < 0.05.

The PAPmean correlated with all respiratory parameters (respiratory rate (Rr), p${}_{\mathrm{et}}$CO${}_{2}$, p${}_{\mathrm{et}}$O${}_{2}$, VE/VCO${}_{2}$, VE/VO${}_{2}$, and VE). Considering the exercise levels, these correlations improved with increasing exercise levels from rest via unloaded cycling to 25 W. Aside from Rr, the correlations with PAPmean were consistent at both resting positions (supine and 45°). The pressures RAPmean and PAWP did not correlate with any of these parameters, except at 25 W (Figure 3). PVR correlated with none of these respiratory parameters except respiratory rate (at unloaded cycling with r = 0.18; at 25 W with r = 0.27; both *p* ≤ 0.05) and inversely with the breathing reserve in percent (%BR, from r = −0.40 at rest to −0.45 at 25 W, all *p* < 0.001). As above, there were no correlations at 50 W.

## 4. Discussion

Our study proved significant correlations (a) between VO${}_{2}$/body weight and CI and (b) between all respiratory parameters and PAPmean. These correlations remained significant at supine rest, 45° rest, unloaded cycling and 25 W exercise. To our knowledge, such a constant relationship through different levels of rest and exercise has not been shown before. Aside from the pathopysiological causality, it was necessary to apply our method with a maximum synchronization of CO, ventilatory parameters and blood gas measurements to unravel these correlations. The literature on iCPET covers a large spectre of assessed parameters but left relevant gaps. In a recent review [3], we identified 53 studies, of which only 7 studies included more than 100 patients. None of these studies applied both methods of CO measurement (TD and dFM), and the included exercise variables were limited. Comparably to [3], the method of iCPET has been summarised by the Boston working group in 2013 [21] and recommendations on measuring CO during exercise have been updated in recent years [10]. Following these recommendations, iCPET has a central role in the advanced work-up of dyspnea and the according diagnostic algorithms [11]. However, these recommendations do not refer to correlations between gas exchange and exercise hemodynamics.

### 4.1. iCPET and Correlations

Correlations have been shown for defined patient groups and mostly at peak exercise level. In a very early study on 23 COPD patients, Schrijen and co-workers found a slight correlation between peakVO${}_{2}$ and hemodynamics (namely PVR) at rest and during light exercise [22]. In patients with chronic heart failure, the working group around Marco Metra published correlations in 1992: they showed that in 33 patients, peakVO${}_{2}$ and VE/VCO${}_{2}$-slope correlated with CI and PVR but not with PAPmean [12]. Some years later and based on a larger number of patients (*n* = 219) this group could also give an equation to calculate CO from peakVO${}_{2}$ as follows: CO = 0.00625 · VO${}_{2}$ + 1.51 [6]. The application of iCPET was extended to patients with connective tissue diseases [13], systemic sclerosis [23], and hypertrophic cardiomyopathy [24]. The latter study showed interesting correlations between PAPmean and the VE/VCO${}_{2}$-ratio at peak exercise and p${}_{\mathrm{et}}$CO${}_{2}$ at anaerobic threshold, but there was no correlation to CO, and with a time delay of at mean 3 days between RHC and CPET, this is not strictly comparable to an iCPET protocol.

Another not literally iCPET study combined consecutively performed CPET and RHC in patients with systemic sclerosis and found that peakVO${}_{2}$, nadir VE/VCO${}_{2}$, and peak p${}_{\mathrm{et}}$CO${}_{2}$ correlate with PAPmean, TPG and PVR at rest [23]. The potential value of iCPET for the discrimination of combined pre- and postcapillary PH and isolated post-capillary PH was shown in patients with chronic heart failure (CHF). In this group, PAPmean correlated again with VE/VCO${}_{2}$, TPG, and also with the dead space/tidal volume ratio (V${}_{d}$/V${}_{t}$) [25]. Recently, two great studies from the Boston working group proved the prognostic value of iCPET. Based on the physiological coupling of PAPmean to the corresponding increase in CO, they assessed 714 patients with exertional dyspnea and proved that the PAPmean/CO-slope was predictive for survival. A cut-off of PAPmean/CO-slope > 3 mmHg/L/min separated all respiratory parameters. Correlations with hemodynamic parameters were not the aim of this study and were not published [8]. The second study with 493 patients and exertional dyspnea was restricted to HFpEF and revealed, comparably to the systemic sclerosis study mentioned above, that VE/VCO${}_{2}$ at its nadir is a decisive parameter for prognosis and associated with CO, PVR and PAPmean/CO-slope [7].

### 4.2. Physiological Considerations

Studies that assessed iCPET at a low level exercise are rare. In an older small study (*n* = 10) on pulmonary hypertension, PAPmean correlated with VE/VCO${}_{2}$ at 30 W but not with VO${}_{2}$ [13]. The Boston/Yale working group showed that in PH patients, the RV function deteriorates already at 50% of peak oxygen uptake. This is in line with our finding that PAPmean correlates with many respiratory parameters at unloaded cycling and even better at 25 W. However, a direct comparison to our study is prevented by their use of a steeper exercise protocol and elaborated computing algorithms [26]. Under physiological conditions, PVR must decrease at exercise to cushion the up to fivefold increase in right ventricular output [11]. This may explain the prognostic cut-off for the PAPmean/CO-slope > 3 mmHg/L/min [8]. A late rise in PAPmean beyond the anaerobic threshold (“takeoff-type”) is associated with higher CO and peakVO${}_{2}$ despite no differences in PAPmean at peak exercise [27].

Inequalities between ventilation and perfusion make gas exchange less efficient than normal and increase the physiological dead space and the V${}_{d}$/V${}_{t}$ ratio, respectively. This leads to a lower p${}_{\mathrm{et}}$CO${}_{2}$ at rest and exercise, as shown in patients with PAH [28] and CHF [29]. Lactate acidosis at already low work rates is a second factor that might lead to an increased ventilatory drive and consecutively increased dead space ventilation and lowered p${}_{\mathrm{et}}$CO${}_{2}$. Hence the decrease of p${}_{\mathrm{et}}$CO${}_{2}$ was more marked in patients with more severe heart failure [29]. This increased V${}_{d}$/V${}_{t}$ ratio might explain the correlation of lower respiratory reserve and higher PVR in our study, as well as the correlation of respiratory rate and PVR. By contrast, a study on COPD and PH (Nice class 3) found that a higher PAPmean was associated with a higher breathing reserve [30]. This illustrates, in our opinion, the shift from a strict pulmonary to a pulmonary-circulatory limitation of exercise.

In an attempt to find an alternative test for subjects who are unable to exercise, a very recent study compared the pulmonary vascular distensibility measured by iCPET with the distensibility after passive leg raise [31]. This study found a correlation of both methods for distensibility, but we still consider iCPET as indispensable because this correlation becomes blurred if the healthy control subjects in this study are omitted. This is in line with our own findings [16] that exercise and fluid challenge activate different pathophysiological pathways and unmask diastolic dysfunction in different patient groups.

## 5. Strengths of the Study

This is the first greater study that assessed iCPET at different and lower levels of exercise. It studies a typical cohort of patients with unexplained dyspnea and typical comorbidities. It provides robust correlations between hemodynamic and respiratory parameters at different exercise levels. Moreover, the study minimized the time delay between hemodynamic and respiratory measurement. Further shortening of the respiratory interval would make the measurement susceptible for outliers in the form of shallow or sigh breathing.

## 6. Limitations

We did not calculate the new and prognostic parameter PAPmean/CO-slope. The study took more than 4 years, and a new definition of PH was presented in the middle of this time. A further evaluation of the prognostic value of iCPET at low-level exercise will require the application of this defintion.

## 7. Conclusions

Our study shows that in dyspnea patients of different etiologies, the cardiac index is closely linked to VO${}_{2}$ at every level of rest and submaximal exercise. PAPmean is the only pressure that correlates with different respiratory parameters, but this correlation is highly significant and stable at rest, unloaded cycling and at 25 W.

## Figures and Tables

**Table 1:** **Characteristics of the study population.** Continuous data are expressed as median and 25th and 75th percentile in brackets; categorical data as absolute number and percentages.

Parameter	Patients	Percent	Median (25th; 75th)
	*n* = 146		
female	72	49.3	
age		(range 22–85)	69 (59; 76)
height (cm)			170 (162; 176)
weight (kg)			80 (68; 98)
BMI (kg/m²)			27.2 (24.1; 32.4)
**Diagnoses leading to iCPET**			
HFpEF	44	30.1	
CTD including systemic sclerosis	25	17.1	
PAH (suspected echocardiography)	25	17.1	
pulmonary embolism	20	13.7	
interstitial lung disease	6	4.1	
COPD	5	3.4	
HFrEF	5	3.4	
congenital heart disease	5	3.4	
dyspnea of unknown orign	4	2.7	
other with *n* ≤ 2 ${}^{\left(1\right)}$	7	4.8	
**Comorbidities**			
(double categorization possible)			
arterial hypertension	97	66.4	
atrial fibrillation	45	30.8	
diabetes mellitus	42	28.8	
venous thrombembolism	41	28.1	
coronary heart disease	37	25.3	
history of cancer	26	17.8	
chronic renal failure	24	16.4	
peripheral arterial obstruction	3	2.1	
**Echocardiography**	135	92.5	
LVEF 35–40%	2	1.5	
LVEF ≥ 50%	133	98.5	
TAPSE (mm)	116		
TR ≥ 1°	81	60.0	
RV${}_{\mathrm{sys}}$ estimated (mmHg)	92		
diastolic dysfunktion	55	40.7	
**Right heart catheterization at rest**			
RAPmean (mmHg)	144		8.0 (6.0; 11.0)
PAPmean (mmHg)	146		28.0 (20.0; 40.0)
PAPmean ≥ 25 mmHg ${}^{\left(2\right)}$	83	56.8	
PAWP (mmHg)	144		14.0 (10.0; 19.0)
PAWP < 15 mmHg			
PVR (WU) TD	144		2.34 (1.32; 3.90)
CO (l/min) TD	145		5.00 (4.28; 6.01)
CI (l/min/m²) TD	145		2.61 (2.28; 3.02)
PVR (WU) iFM	143		2.68 (1.47; 4.74)
CO (l/min) iFM	145		4.30 (3.64; 5.05)
CI (l/min/m²) iFM	144		2.25 (1.98; 2.57)
PVR (WU) dFM	140		2.24 (1.20; 3.52)
CO (l/min) dFM	141		5.55 (4.34; 6.73)
CI (l/min/m²) dFM	141		2.88 (2.45; 3.49)
**Pulmonary function**			
TLC (% predicted) ${}^{\left(3\right)}$	132		93.4 (81.9; 104.4)
reduced < 80%	31	23.5	
FVC (% predicted)	137		92.3 (5.5; 104.5)
reduced < 80%	40	29.2	
FEV1 (% predicted)	137		86.1 (69.4; 97.9)
FEV1/FVC (%)	137		75.8 (69.9; 80.2)
RV (% predicted)	132		107.6 (87.1; 121.0)
RV/TLC (% predicted)	131		44.8 (38.3; 50.9)
DLCO${}_{c}$ (% predicted)	122		59.8 (44.1; 72.2)
Reduced < 60%	62	50.8	
KCO (% predicted)	100		74.2 (56.4; 90.5)
reduced < 60%	28	28.0	
**Cardiopulmonary exercise testing**			
maximum power (Watt)	142		84.0 (52.0; 100.0)
maximum power (% predicted)	142		57.3 (43.7; 69.1)
peakVO${}_{2}$ (ml/min/kg)	139		13.5 (11.3; 17.6)
peakVO${}_{2}$ (% predicted) ${}^{\left(3\right)}$	142		63.6 (51.7; 75.4)
peakVO${}_{2}$/HR (ml)	142		10.0 (8.0; 12.8)
VE/VCO${}_{2}$-slope	142		40.0 (32.0; 49.0)
increased > 34	98	69.0	
VE/VCO${}_{2}$ @rest	138		41.0 (5.7; 47.7)
VE/VCO${}_{2}$ @AT	134		38.2 (32.6; 44.5)
p${}_{\mathrm{et}}$CO${}_{2}$ @rest (mmHg)	142		27.1 (23.3; 30.5)
p${}_{\mathrm{et}}$CO${}_{2}$ @AT	136		29.0 (25.1; 33.5)
peak AaDO${}_{2}$ (mmHg)	141		40.5 (31.0; 56.2)
increased > 35 mmHg	93	66.0	
p${}_{a-\mathrm{et}}$CO${}_{2}$ (mmHg)	141		7.2 (4.4; 10.2)
VE/MVV (%)	137		63.1 (49.8; 73.7)
increased > 80%	13	9.5	
p${}_{a}$O${}_{2}$ @rest (mmHg)	142		70.8 (58.5; 77.4)
p${}_{a}$O${}_{2}$ @peak (mmHg)	141		68.1 (54.4; 81.5)
p${}_{a}$CO${}_{2}$ @rest (mmHg)	142		35.0 (31.9; 37.8)
p${}_{a}$CO${}_{2}$ @peak (mmHg)	141		34.7 (31.2; 38.7)

AaDO_2_ difference of arterial and end tidal pressure of oxygen, AT anaerobic threshold, BMI body mass index, CI cardiac index, CO cardiac output, COPD chronic obstructive pulmonary disease, CTD connective tissue disease, DLCO diffusion capacity of carbon monoxide, ILD interstitial lung disease, KCO Krogh factor (DLCO per alveolar volume), CPET cardiopulmonary exercise testing, dFM direct method of Fick, FEV1 forced expiratory volume in one second, FVC forced vital capacity, HFpEF heart failure with preserved ejection fraction, HR heart rate, HFrEF heart failure with reduced ejection fraction, iFR indirect method of Fick, IQR interquartile range, LVEF left ventricular ejection fraction, PAH pulmonary arterial hypertension, PAPmean mean pulmonary arterial pressure, p_a_CO_2_ arterial partial pressure of carbon dioxide, p_a-et_CO_2_ difference of capillary and end tidal pressure of carbon dioxide, p_a_O_2_ arterial partial pressure of oxygen, peakVO_2_ maximum oxygen uptake, PH pulmonary hypertension, PVR pulmonary vascular resistance, RAPmean right atrial pressure, RV residual volume, RV_sys_ calculated right ventricular pressure by echocardiography, TAPSE tricuspidal annular plane systolic excursion, TD thermodilution, TLC total lung capacity, TR tricuspidal valve regurgitation, VE/MVV ratio of ventilation to maximum voluntary ventilation, VE/VCO_2_@AT ratio of ventilation to carbon dioxide output at anaerobic threshold, VE/VCO_2_-slope slope of the relation between ventilation and carbon dioxide output, WU wood units. ^(1)^ other diagnoses leading to iCPET: Obstructive sleep apnea syndrom *n* = 1, portopulmonal hypertension *n* = 2, chronic pulmonary fibrosis with emphysema *n* = 2, sarkoidosis = 1, obesitas hypoventilation syndrome = 1; ^(2)^ The recent definition with a cutoff at PAPmean > 20 mmHg was published 2019 and therefore not applicable for our study that started 2016. See please in the methods section of the manuscript; ^(3)^ Reference values for pulmonary function see please ref. [19] and for CPET parameters ref. [20].

## Data Availability

Data are available on request at the corresponding author.

## Abbreviations

AaDO${}_{2}$	arterial-alveolar pressure difference for oxygen
CI	cardiac index
CHF	chronic heart failure
CO	cardiac output
CPET	cardiopulmonary exercise testing
iCPET	invasive cardiopulmonary exercise testing
HFpEF	heart failure with preserved ejection fraction
HFrEF	heart failure with reduced ejection fraction
p${}_{a}$CO${}_{2}$	arterial partial pressure of carbon dioxide
p${}_{a}$O${}_{2}$	arterial partial pressure of oxygen
p${}_{\mathrm{et}}$CO${}_{2}$	end-tidal partial pressure of carbon dioxide
p${}_{\mathrm{et}}$O${}_{2}$	end-tidal partial pressure of oxygen
PAH	pulmonary arterial hypertension
PAPmean	mean pulmonary artery pressure
PAWP	pulmonary arterial wedge pressure
peakVO${}_{2}$	maximum oxygen uptake per minute at exercise
PH	pulmonary hypertension
PVR	pulmonary vascular resistance
RER	respiratory exchange ratio
RHC	right heart catheterisation
Rr	respiratory rate
RV${}_{\mathrm{sys}}$	right ventricular systolic pressure
SD	standard deviation
TD	thermodilution
V${}_{d}$	physiological dead space
VE	ventilation per minute
V${}_{t}$	tidal volume
VE/VCO${}_{2}$	ventilation to carbon dioxide output ratio
VE/VO${}_{2}$	ventilation to oxygen uptake ratio
VE/VCO${}_{2}$-slope	slope of the relation of ventilation and carbon dioxide output, ventilatory efficiency
VO${}_{2}$	oxygen uptake per minute
WU	Wood unit
